# An induced pluripotent stem cell line (TRNDi010-C) from a patient carrying a homozygous p.R401X mutation in the *NGLY1* gene

**DOI:** 10.1016/j.scr.2019.101496

**Published:** 2019-07-09

**Authors:** Shu Yang, Yu-Shan Cheng, Rong Li, Manisha Pradhan, Junjie Hong, Jeanette Beers, Jizhong Zou, Chengyu Liu, Matt Might, Steven Rodems, Wei Zheng

**Affiliations:** a National Center for Advancing Translational Sciences, National Institutes of Health, Bethesda, MD, USA; b iPSC core, National Heart, Lung and Blood Institute, National Institutes of Health, Bethesda, MD, USA; c Transgenic Core, National Heart, Lung and Blood Institute, National Institutes of Health, Bethesda, MD, USA; d University of Alabama at Birmingham, Birmingham, AL, USA; e Retrophin, Inc., San Diego, CA, USA

## Abstract

NGLY1 deficiency is a rare inherited disorder caused by mutations in the *NGLY1* gene encoding N-glycanase 1 that is a hydrolase for N-linked glycosylated proteins. An induced pluripotent stem cell (iPSC) line was generated from the dermal fibroblasts of a 16-year-old patient with homozygous mutation of p.R401X (c.1201 A >T) in the NGLY1 gene. Our iPSC model offers a useful resource to study the disease pathophysiology and to develop therapeutics for treatment of NGLY1 patients.

## Resource utility

This TRNDi010-C iPSC line presents a patient-specific disease model for studies of NGLY1 deficiency phenotype and pathophysiology and can be used as a cell-based model for drug discovery and therapeutic development to treat NGLY1 patients.

## Resource details

NGLY1 deficiency, also known as NGLY1-related congenital disorder of deglycosylation, is a rare autosomal recessive disorder caused by mutations in the *NGLY1* gene which encodes a specialized enzyme called N-glycanase that removes N-linked glycan from glycosylated proteins within the body. Deficiency of this protein can lead to malfunctions of cellular functions and accumulation of misfolded proteins within cells in specific tissues or organs. The symptoms and severity of this disease can dramatically vary among affected individuals, who may have developmental delays, intellectual disability, movement disorders, seizures, liver disease, and alacrima (Enns et al., 2014; Lam et al., 2017; Caglayan et al., 2015).

**Fig. 1.**
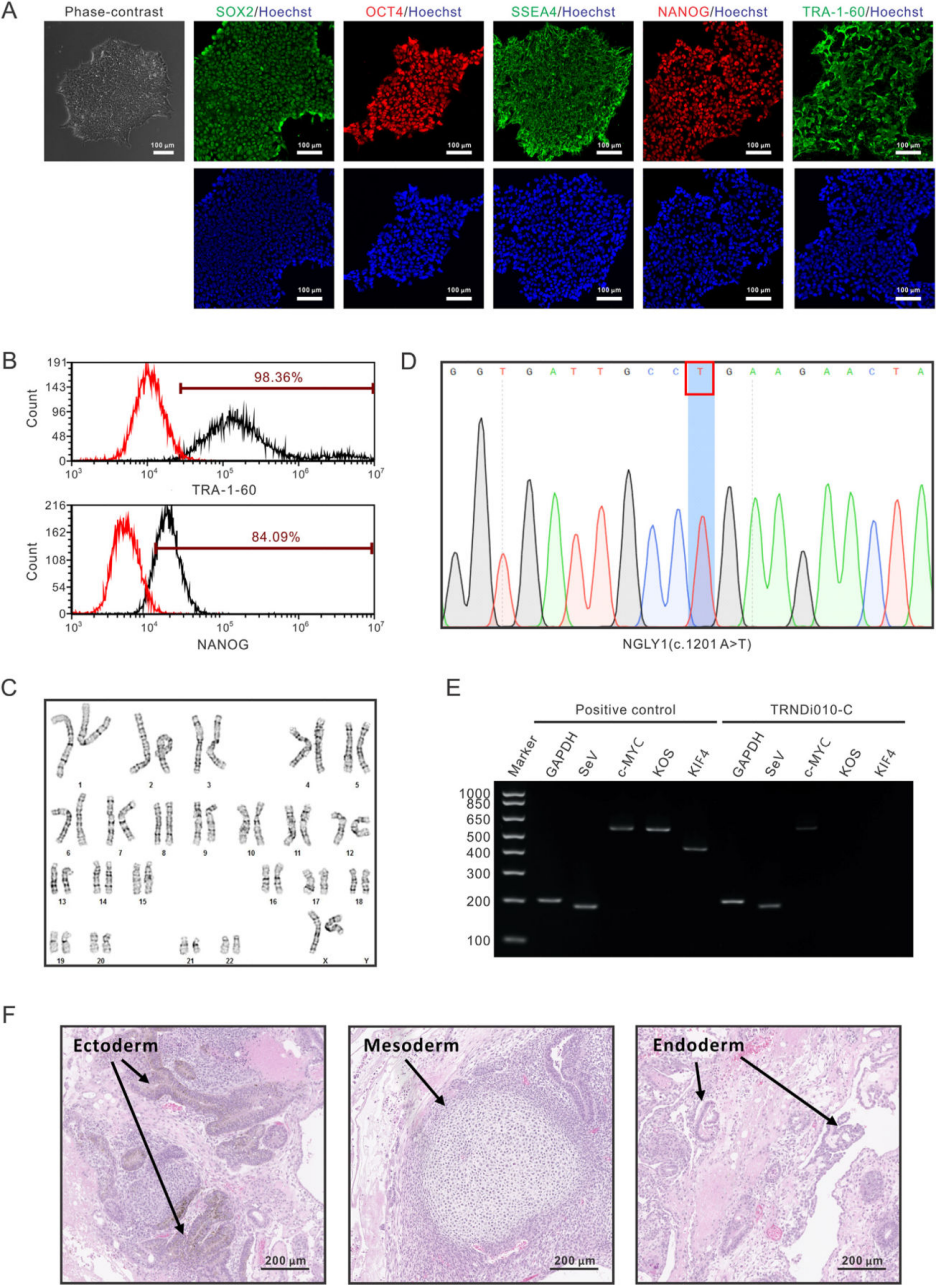


In this study, a human dermal fibroblast was derived from a 16-year-old female patient (GM26612, Coriell Institute) with a homozygous nonsense mutation of p.R401X (c.1201A > T) in exon 8 of the *NGLY1* gene (3p24.2) (Enns et al., 2014; Need et al., 2012). The iPSC line, TRNDi010-C, was reprogramed from the fibroblasts using the non-integrating CytoTune-Sendai viral vector kit (A16517, Thermo Fisher Scientific) containing four pluripotency transcription factors, OCT3/4, KLF4, SOX2, and c-MYC (Beers et al., 2015). Individual colonies were picked, expanded and further analyzed at the cellular and genetic level to confirm successful reprogramming (Table 1). The TRNDi010-C iPS cells displayed the standard pluripotent stem cell morphology under phase contrast microscopy and expressed pluripotency markers OCT4, NANOG and SOX2 in the nuclei and SSEA4 and TRA-1–60 on the plasma membrane (Fig. 1A). The quantitative analysis by flow cytometry revealed 98.36% and 84.09% expression rate of TRA-1–60 and NANOG, respectively (Fig. 1B). G-banded karyotyping analysis was used to confirm the karyotype, which showed the normal diploid 46, XX, without any detectable abnormalities (Fig. 1C). The genetic mutation, c.1201A > T (p.R401X), was validated by Sanger sequencing of the PCR product harboring the single nucleotide variant (Fig. 1D), consistent with the description of Coriell Institute. After passage 30, the exogenous reprogramming factors were eliminated from TRNDi010-C iPSCs, despite the remaining low level of SeV (Fig. 1E). To further test the pluripotency of this iPSC line, teratoma formation experiment was performed. As shown in Fig. 1F, the imaging data identified its ability to generate derivative of three germ layers, ectoderm, mesoderm and endoderm *in vivo*. Furthermore, this iPSC was negative for mycoplasma contamination (Supplementary Fig. S1). The STR DNA profile of the TRNDi010-C matched with its parental GM26612 fibroblast at all 18 loci (information available with the authors).

## Materials and methods

### Cell culture and reprogramming

Patient skin fibroblasts were obtained from Coriell Cell Repositories (GM26612), and cultured in DMEM supplemented with 10% fetal bovine serum, 100 units/mL penicillin and 100 μg/mL streptomycin in a humidified incubator with 5% CO_2_ at 37 °C. Patient fibroblasts were reprogrammed into iPSCs using the non-integrating Sendai virus technology (Beers et al., 2015). Human iPSCs were cultured in mTeSR™1 (STEMCELL Technologies) on Matrigel (Corning, 354277)-coated plates at 37 °C in humidified air with 5% CO_2_ and 5% O_2_. The cells were passaged with ReLeSR™ (STEMCELL Technologies) at generally 1:10 ratio when they reached around 70% confluency.

### Genome analysis

The genome analysis of variants in NGLY1 was conducted through Applied StemCell (Milpitas, California, USA). Briefly, genomic DNA was extracted from iPSC line TRNDi010-C using QuickExtract™ DNA Extraction Solution (Lucigen) followed by PCR amplification using MyTaq™ Red Mix (Bioline, Taunton, MA). Amplifications were carried out on T00 Thermal Cycler from Bio-Rad (#1861096) using the following program: 95 °C, 2 min; 35 cycles of [95 °C, 15 s; 60 °C, 15 s; 72 °C, elongation duration varies by amplicon size], 72 °C 5 min; 4 °C, indefinite. Genotyping of the homozygous for the p.R401X variant (c.1201 A > T) in exon 8 of the *NGLY1* gene was performed using Sanger sequencing analysis. The specific primers for gene amplification and sequencing are listed in Table 2.

### Immunocytochemistry

iPSC colonies, cultured in the 96-well plate, were washed with Dulbecco’s phosphate-buffered saline (DPBS) without Ca^2+^ and Mg^2+^ and fixed in 4% paraformaldehyde for 15 min at room temperature. Fixed cells were washed with DPBS twice, and permeabilized with 0.1% Triton X-100 in DPBS for 15 min. After 1 h of blocking, the cells were incubated with primary antibodies, diluted in the blocking buffer, for overnight at 4 °C. Cells were washed twice with DPBS and a corresponding secondary antibody conjugated with Alexa Fluor 488 or Alex Fluor 647 was added to the cells and incubated for 1 h at room temperature (Antibodies used are listed in Table 2). Cells were then stained with Hoechst 33342 for 15 min and imaged using an INCell Analyzer 2500 imaging system (GE Healthcare) with Cy5, FITC and DAPI filter sets.

### Flow cytometry analysis

The iPSCs were dissociated by TrypLE Express enzyme (Thermo Fisher Scientific). After washing once with DPBS, cells were fixed with 4% paraformaldehyde for 10 min and were permeabilized with 0.2% Tween-20 in DPBS for another 10 min at room temperature, followed by staining with fluorophore-conjugated antibodies (Table 2) for 1 h at 4 °C. The cells were then analyzed on a BD AccuriC6 FlowCytometry system (BD Biosciences).

### G-banded karyotyping

The G-banded karyotyping analysis was performed by the WiCell Research Institute (Madison, WI) using the iPS cells at passage 6. Twenty randomly selected metaphase cells were selected for the standard cytogenetic analysis.

### Short tandem repeat (STR) analysis

The STR analysis of patient fibroblasts and iPSCs was performed by the Johns Hopkins University Genetic Resources Core Facility using the Promega PowerPlex 18D Kit. The ABI Prism® 3730xl Genetic Analyzer was used to electrophorese the PCR products and GeneMapper® v 4.0 software (Applied Biosystems) was used to analyze the data.

### Mycoplasma detection

The Lonza MycoAlert kit was used to assess the mycoplasma according to the instructions from the company. B/A ratio > 1.2 indicates the positive sample; 0.9–1.2 indicates the ambiguous result; < 0.9 indicates the negative sample.

### Sendai virus detection

Using the RNeasy Plus Mini Kit (Qiagen), the total RNA was extracted. The cDNA was reverse-transcribed from 1 μg RNA by SuperScript™ III First-Strand Synthesis SuperMix (Thermo Fisher Scientific). The Platinum II Hot-Start PCR Master Mix (Thermo Fisher Scientific) was used to amplify the target sequence with a PCR program: 94 °C, 2 min; 30 cycles of 94 °C, 15 s, 60 °C, 15 s, and 68 °C, 15 s on Mastercycler pro S (Eppendorf) with the specific primers (Table 2). The human fibroblasts (GM05659, Coriell Institute) transfected with Sendai virus for 4 days was used as the positive control.

### Teratoma formation assay

Patient iPSCs were dissociated with 0.5 mM EDTA in PBS and were resuspended approximately 1 × 10^7^ cells in 400 μL culture medium supplied with 25 mM HEPES (pH 7.4) and stored on ice. Then, 50% volume (200 μL) of cold Matrigel (Corning, 354277) was added and mixed with the cells. The mixture was injected subcutaneously into NSG mice (JAX No. 005557) at 150 μL per injection site. Visible tumors were removed 6–8 weeks post-injection and were immediately fixed in 10% Neutral Buffered Formalin. The fixed tumors were embedded in paraffin and stained with hematoxylin and eosin.

## Supplementary Material

1

## Figures and Tables

**Table 1:** Characterization and validation.

Classification	Test	Result	Data
Morphology	Photography	Normal	Fig. 1 Panel A
Phenotype	Immunocytochemistry	SOX2, OCT4, NANOG, SSEA-4, TRA-1–60	Fig. 1 Panel A
	Flow cytometry	TRA-1–60 (98.36%); NANOG (84.09%)	Fig. 1 Panel B
Genotype	Karyotype (G-banding) and resolution	46XX Resolution: 350–400	Fig. 1 Panel C
Identity	Microsatellite PCR (mPCR) OR	Not performed	N/A
	STR analysis	18 sites tested, all sites matched	Available with the authors
Mutation analysis (IF APPLICABLE)	Sequencing	Homozygous mutation *NGLY1*, p.R401X	Fig. 1 Panel D
	Southern Blot OR WGS	N/A	N/A
Microbiology and virology	Mycoplasma	Mycoplasma testing by luminescence. Negative	Supplementary Fig. S1
Differentiation potential	Teratoma formation	Teratoma with three germ layers formation, ectoderm, mesoderm and endoderm.	Fig. 1 Panel F
Donor screening (OPTIONAL)	HIV 1 + 2 Hepatitis B, Hepatitis C	N/A	N/A
Genotype additional info (OPTIONAL)	Blood group genotyping	N/A	N/A
	HLA tissue typing	N/A	N/A

**Table 2:** Reagents details.

Antibodies used for immunocytochemistry/flow-cytometry			

	Antibody	Dilution	Company Cat # and RRID

Pluripotency Markers	Mouse anti-SOX2	1:50	R & D systems, Cat# MAB2018, RRID: AB_358009
Pluripotency Markers	Rabbit anti-NANOG	1:400	Cell signaling, Cat# 4903, RRID: AB_10559205
Pluripotency Markers	Rabbit anti-OCT4	1:400	Thermo Fisher, Cat# A13998, RRID: AB_2534182
Pluripotency Markers	Mouse anti-SSEA4	1:1000	Cell signaling, Cat# 4755, RRID: AB_1264259
Pluripotency Markers	Mouse anti- TRA-1–60	1:1000	Cell signaling, Cat# 4746, RRID: AB_2119059
Secondary Antibodies	Donkey anti-Mouse IgG (Alexa Fluor 488)	1:400	Thermo Fischer, Cat# A21202, RRID: AB_141607
Secondary Antibodies	Donkey anti-Rabbit IgG (Alexa Fluor 594)	1:400	Thermo Fischer, Cat# A21207, RRID: AB_141637
Flow Cytometry Antibodies	Anti-Tra-1–60-DyLight 488	1:50	Thermo Fischer, Cat# MA1–023-D488X, RRID: AB_2536700
Flow Cytometry Antibodies	Anti-Nanog-Alexa Fluor 488	1:50	Millipore, Cat# FCABS352A4, RRID: AB_10807973
Flow Cytometry Antibodies	Mouse-IgM-DyLight 488	1:50	Thermo Fischer, Cat# MA1–194-D488, RRID: AB_2536969
Flow Cytometry Antibodies	Rabbit IgG-Alexa Fluor 488	1:50	Cell Signaling, Cat# 4340S, RRID: AB_10694568

Primers		

	Target	Forward/Reverse primer (5′–3′)

Sev specific primers (RT-PCR)	Sev/181 bp	F: GGA TCA CTA GGT GAT ATC GAG C R: ACC AGA CAA GAG TTT AAG AGA TAT GTA TC
Sev specific primers (RT-PCR)	KOS/528 bp	F: ATG CAC CGC TAC GAC GTG AGC GC R: ACC TTG ACA ATC CTG ATG TGG
Sev specific primers (RT-PCR)	Klf4/410 bp	F: TTC CTG CAT GCC AGA GGA GCC C R: AAT GTA TCG AAG GTG CTC AA
Sev specific primers (RT-PCR)	C-Myc/523 bp	F: TAA CTG ACT AGC AGG CTT GTC G R: TCC ACA TAC AGT CCT GGA TGA TGA TG
House-Keeping gene (RT-PCR)	GAPDH/197bp	F: GGA GCG AGA TCC CTC CAA AAT R: GGC TGT TGT CAT ACT TCT CAT GG
Targeted mutation analysis (PCR)	NGLY1 (c.1201A > T)/258bp	F: GAC AAC AGA GCG AGA CTT C R: AAA AAG ATA GCC ACA CCA TAC C

**Key resources table:** 

Unique stem cell line identifier	TRNDi010-C
Alternative name(s) of stem cell line	HT592C
Institution	National Institutes of Health National Center for Advancing Translational Sciences Bethesda, Maryland, USA
Contact information of distributor	Dr. Wei Zheng, Wei.Zheng@nih.gov
Type of cell line	iPSC
Origin	Human
Additional origin info	Age: 16-year-old Sex: Female Ethnicity: Caucasian
Cell Source	Skin fibroblasts
Clonality	Clonal
Method of reprogramming	Integration-free Sendai viral vectors
Genetic Modification	Yes
Type of Modification	Hereditary
Associated disease	NGLY1 Deficiency
Gene/locus	NGLY1^R401X^
Method of modification	N/A
Name of transgene or resistance	N/A
Inducible/constitutive system	N/A
Date archived/stock date	04–23–2018
Cell line repository/bank	N/A
Ethical approval	NIGMS Informed Consent Form was obtained from patient at time of sample submission. Confidentiality Certificate: CC-GM-15–004
