# Supplementary figures and images for: An induced pluripotent stem cell line (TRNDi010-C) from a patient carrying a homozygous p.R401X mutation in the NGLY1 gene
Source: Stem Cell Res. Author manuscript; Available in PMC 2019 Dec 13. (PMC6910241; doi:10.1016/j.scr.2019.101496)

## Slide 1
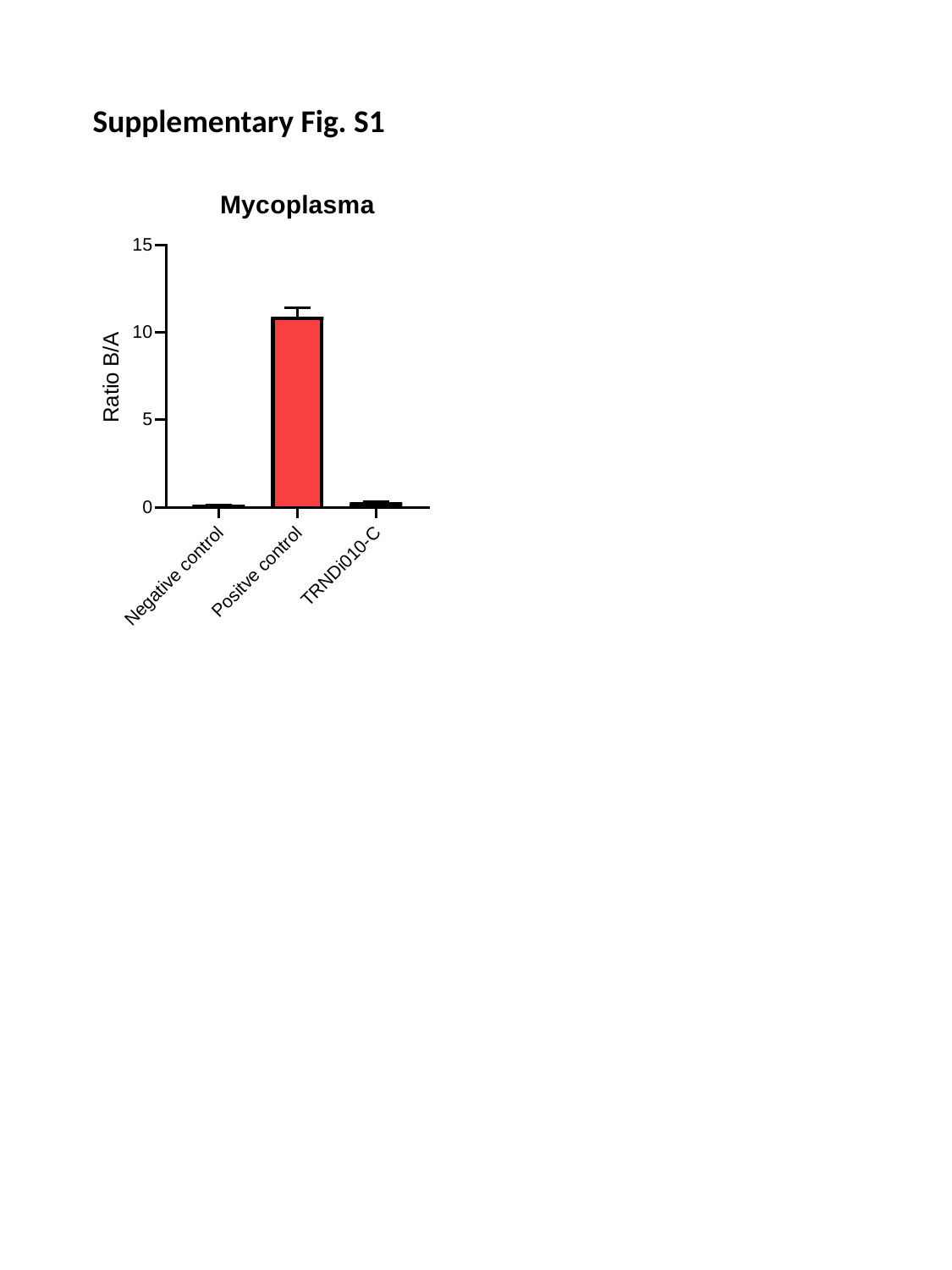

Supplementary Fig. S1

Supplement: 1 [file NIHMS1544338-supplement-1.pptx]
